# Essential Updates 2023/2024: Recent Advances of Multimodal Approach in Patients for Gastric Cancer

**DOI:** 10.1002/ags3.70041

**Published:** 2025-05-14

**Authors:** Katsutoshi Shoda, Yoshihiko Kawaguchi, Suguru Maruyama, Daisuke Ichikawa

**Affiliations:** ^1^ First Department of Surgery, Faculty of Medicine University of Yamanashi Yamanashi Japan

**Keywords:** conversion surgery, immune checkpoint inhibitors, minimally invasive surgery, neoadjuvant chemotherapy, perioperative care

## Abstract

Gastric cancer remains a major global health burden, especially in East Asia. Recent years have brought significant advances in multimodal management, including surgery, perioperative chemotherapy, immunotherapy, and supportive care. This review summarizes key updates from 2023 to 2024, focusing on surgical innovations, perioperative systemic therapy, treatment strategies for stage IV disease, and nutritional interventions. In early and locally advanced disease, laparoscopic and robotic gastrectomy have become widely accepted in Asia, supported by large, randomized trials such as JCOG0912 and KLASS‐02. Perioperative chemotherapy has become a global standard, with the FLOT regimen leading in the West and SOX, XELOX, or DOS emerging in Asia. Immunotherapy is increasingly incorporated into perioperative settings, with trials like KEYNOTE‐585 and NEONIPIGA suggesting potential benefit in selected patients. For stage IV gastric cancer, systemic chemotherapy remains the mainstay, but conversion surgery and treatment of oligometastasis have shown promising results in selected cases. Trials such as JCOG1704 and AIO‐FLOT5 are investigating optimal strategies. Perioperative nutrition has also gained attention, with oral nutritional supplements, ERAS protocols, and digital tools like continuous glucose monitoring (CGM) and AI‐driven platforms enhancing outcomes. Looking ahead, integration of molecular biomarkers (e.g., HER2, PD‐L1, MSI, ctDNA), precise staging, and multidisciplinary collaboration will be essential for personalized treatment. Ongoing trials and international cooperation are expected to further refine evidence‐based approaches to gastric cancer care.

## 1 Introduction

Gastric cancer remains one of the leading causes of cancer‐related deaths worldwide, with a particularly high burden in East Asian countries such as Japan and Korea. According to GLOBOCAN 2020, it ranks fifth in incidence and fourth in mortality among all malignancies globally [1]. While improvements in endoscopic detection and curative surgical techniques have enhanced prognosis in early‐stage disease, advanced‐stage gastric cancer continues to carry a poor prognosis due to factors including late diagnosis, aggressive tumor biology, and limited efficacy of systemic therapies [2].

Recent advancements in the understanding of tumor biology, including molecular subtypes and immune profiling, have catalyzed efforts to personalize treatment strategies [3]. This has led to the development of multimodal approaches that combine surgery with perioperative chemotherapy, targeted agents, and more recently, immunotherapy [4]. The integration of supportive care measures such as perioperative nutrition, physical prehabilitation, and early recovery protocols has also demonstrated benefits in enhancing patient outcomes [5].

There are marked differences in treatment strategies between Eastern and Western countries. In Western populations, perioperative chemotherapy has been established as standard practice for locally advanced disease, primarily based on findings from the MAGIC and FLOT4‐AIO trials [6, 7]. In contrast, Asian practice has emphasized upfront D2 gastrectomy followed by adjuvant chemotherapy, supported by the ACTS‐GC and CLASSIC trials [8, 9]. However, the landscape is shifting as new Asian trials—such as PRODIGY, RESOLVE, and RESONANCE‐II—explore the benefits of neoadjuvant or perioperative approaches [10, 11, 12].

The present review aims to summarize essential updates from 2023 and 2024 on the multimodal management of gastric cancer. It will focus on recent advances in surgical techniques, perioperative systemic therapy, treatment strategies for stage IV disease, and supportive care including nutrition. Emphasis will be placed on regional variations in clinical practice, emerging technologies such as AI‐assisted surgery and digital monitoring tools, and future directions aimed at improving long‐term outcomes and quality of life for patients with gastric cancer.

## 2 Current Status and Prospects of Gastric Cancer Surgery

### 2.1 Early Gastric Cancer

Minimally invasive surgery, particularly laparoscopic gastrectomy, has become the standard treatment for early‐stage gastric cancer in East Asia. Several multicenter randomized controlled trials (RCTs) have established its safety and efficacy. The JCOG0912 trial in Japan demonstrated non‐inferiority in relapse‐free survival for laparoscopic distal gastrectomy compared to open surgery, with advantages in postoperative recovery [13]. Similarly, the Korean KLASS‐01 and the Chinese CLASS‐01 trials confirmed the oncologic safety and reduced morbidity associated with laparoscopic approaches [14].

Sentinel lymph node navigation surgery (SNNS) is being actively investigated as a function‐preserving strategy to limit lymphadenectomy in early gastric cancer. The Korean SENORITA trial demonstrated that laparoscopic SNNS significantly improved postoperative quality of life; however, it failed to confirm non‐inferiority in recurrence‐free survival compared to standard gastrectomy with D2 lymphadenectomy [15]. In Japan, the JCOG0302 trial evaluated the feasibility of SNNS and reported high sensitivity but a non‐negligible false‐negative rate, leading to a cautious clinical stance [16]. However, ongoing Japanese trials are now aiming to refine the technique, standardize indications, and re‐evaluate oncologic safety in a multicenter setting [17]. These studies emphasize the potential of SNNS to become a viable option for selected patients, provided technical proficiency and intraoperative pathology protocols are well‐established. The successful integration of SNNS into routine practice will depend on accumulating long‐term evidence, surgical standardization, and advances in intraoperative navigation.

Artificial intelligence (AI) is increasingly integrated into early gastric cancer surgery [18]. AI‐assisted endoscopy aids in tumor detection and margin identification, while machine learning tools are being developed for surgical navigation and intraoperative risk assessment. Although clinical integration is still evolving, pilot studies from Japan and Korea have demonstrated feasibility and growing interest [19].

### 2.2 Resectable Locally Advanced Gastric Cancer

Gastrectomy with D2 lymphadenectomy remains the cornerstone of treatment for locally advanced gastric cancer. The KLASS‐02 and JLSSG0901 trials have established the oncologic safety and non‐inferiority of laparoscopic gastrectomy in this setting [20, 21]. In parallel, robotic gastrectomy has gained increasing interest due to its potential to enhance surgical precision and reduce perioperative complications. Its application in technically challenging areas such as peripancreatic lymphadenectomy is under active investigation. Ongoing clinical trials aim to elucidate whether robotic approaches can offer tangible advantages over conventional laparoscopy in terms of safety, efficacy, and oncologic outcomes.

Robotic‐assisted gastrectomy (RAMIG) is gaining attention for its potential to reduce postoperative complications. Current research, including prospective observational studies, aims to define its benefits and limitations [22].

### 2.3 Gastroesophageal Junction Cancer

Surgery for gastroesophageal junction cancer remains controversial due to anatomical and classification challenges. Siewert type II tumors, which straddle the esophagogastric junction, are treated differently across regions. In Western countries, esophagectomy is favored, while in East Asia, extended total gastrectomy with D2 lymphadenectomy is more common. Ongoing trials such as the CARDIA trial aim to determine the optimal surgical approach. Integration of preoperative imaging and molecular profiling may further guide personalized treatment strategies [23].

## 3 Perioperative Chemotherapy in Gastric Cancer

### 3.1 Differences in Perioperative Strategies: West Versus Asia

Perioperative chemotherapy has become a cornerstone of treatment for locally advanced gastric cancer. However, distinct differences exist between Western and Asian practices. In Western countries, chemotherapy is generally administered both before and after surgery, based on robust evidence from the MAGIC and FLOT trials [6, 24]. In Asia, the standard had long been surgery followed by adjuvant chemotherapy (e.g., S‐1 or XELOX), supported by the ACTS‐GC and CLASSIC trials [8, 9]. More recently, Asian nations have introduced neoadjuvant approaches, exemplified by the RESOLVE and PRODIGY trials [25, 26]. These developments reflect a convergence in multimodal strategies worldwide, although optimal regimens vary based on population characteristics and healthcare systems. Recent major clinical trials of neoadjuvant chemotherapy in gastric cancer are summarized in Table 1.

**TABLE 1:** Summary of recent clinical trials of neoadjuvant chemotherapy in gastric cancer.

Trial (author, year)	Design	No. of patients	Patient cohort	Treatment arms	Primary endpoint	pCR rate (%)	Postop complications (%)	Median OS/HR	References
Tian et al. (2023)	RCT	280	cStage III	Preop DOX	OS	16	NR	HR 0.64	[7]
FLOT4 (2019)	RCT	716	Resectable LAGC	FLOT vs. ECF/ECX	OS	16 vs. 6	55 vs. 55	HR 0.77	[27]
RESONANCE‐II (2020)	RCT	749	cStage II–III	SOX vs. CAPOX	3‐yr DFS	10 vs. 4	NR	NR	[28]
PRODIGY (2021)	RCT	530	cT2–T4N+	Periop DOS	PFS	10	13 vs. 20	HR 0.72	[29]
KEYNOTE‐585 (2024)	RCT	804	HER2‐neg, cStage II–III	pembro + chemo	pCR	13 vs. 2	NR	HR 0.90	[9]
MATTERHORN (2023)	Phase III	474	cStage II/III	durvalumab + FLOT	pCR	19 vs. 7	NR	NR	[10]

Abbreviations: CAPOX, capecitabine and oxaliplatin; DOS, docetaxel, oxaliplatin, and S‐1; DOX, docetaxel, oxaliplatin, and capecitabine; ECF, pirubicin, cisplatin, and infused fluorouracil; ECX, epirubicin, cisplatin and capecitabine; FLOT, fluorouracil, leucovorin, oxaliplatin, and docetaxel; HR, hazard ratio; OS, overall survival; pCR, pathologic complete response; SOX, S‐1 and oxaliplatin.

### 3.2 Western Trials: MAGIC, FLOT4, MATTERHORN

The MAGIC trial first demonstrated a survival benefit for perioperative chemotherapy with ECF (epirubicin, cisplatin, and 5‐FU) versus surgery alone, leading to global interest in multimodal treatment [6]. The FLOT4‐AIO trial later established FLOT (5‐FU, leucovorin, oxaliplatin, and docetaxel) as a superior regimen, increasing R0 resection rates and median OS (50 vs. 35 months, HR 0.77) [30]. The MATTERHORN trial added durvalumab to FLOT and observed an improved pCR rate (19% vs. 7%) with manageable toxicity, particularly in PD‐L1 positive subgroups [27]. These findings have shaped Western standards and stimulated further exploration of combination therapies.

### 3.3 Asian Trials: RESOLVE, PRODIGY, RESONANCE‐II

The RESOLVE trial compared perioperative SOX or XELOX to adjuvant XELOX in over 700 Chinese patients. Results favored the perioperative arms with improved R0 rates (SOX: 89.4%) and 3‐year DFS (SOX: 66.3% vs. XELOX: 60.0%) [25]. The PRODIGY trial confirmed that neoadjuvant DOS followed by surgery and adjuvant S‐1 improved PFS (HR 0.70), particularly in cT3–T4 patients [26]. RESONANCE‐II, a multicenter phase III RCT in Korea, demonstrated improved pCR rates with SOX and enhanced postoperative tolerance, offering further evidence for perioperative treatment in Asian populations [12].

### 3.4 Immunotherapy in the Perioperative Setting

Recent trials have explored the addition of immune checkpoint inhibitors to chemotherapy. KEYNOTE‐585 evaluated pembrolizumab + chemotherapy, reporting an increase in pCR (13% vs. 2%) but no OS difference so far [28]. The NEONIPIGA trial in MSI‐H/dMMR patients showed a 59% pCR rate using neoadjuvant nivolumab and ipilimumab [29]. MATTERHORN trial data are still maturing but show promise in PD‐L1 positive populations. Ongoing studies will clarify the role of immunotherapy, especially in molecularly selected cohorts such as those with high tumor mutation burden or MSI‐H tumors.

### 3.5 Importance of Accurate Staging and Selection

While imaging plays a critical role in staging, its limitations can lead to overtreatment or under‐treatment. In the FLOT4 trial, about 17% of patients clinically staged as cStage II were downstaged to pStage I [7]. The RESOLVE trial observed similar discrepancies [11]. Furthermore, about 2%–3% of patients in neoadjuvant arms experienced progression, making surgery unfeasible. This highlights the need for careful patient selection, pre‐treatment biopsies, and reevaluation after induction therapy using endoscopy and imaging modalities.

### 3.6 Special Considerations in the Elderly

Elderly patients often face challenges in perioperative chemotherapy due to concerns about toxicity and frailty. However, Dutch studies demonstrated that preoperative chemotherapy was tolerable and improved pCR and DFS compared to surgery alone [31]. A cohort of patients aged ≥ 70 receiving FLOT had a pCR of 9%, with no increase in postoperative complications compared to younger patients. Tailored regimens and geriatric assessment tools may optimize outcomes in this population. Real‐world studies are also underway to validate risk stratification tools for this group.

### 3.7 Conclusion and Future Perspectives

Perioperative chemotherapy has evolved into a global standard of care. FLOT remains the benchmark in the West, while Asian countries increasingly adopt neoadjuvant SOX, XELOX, or DOS regimens [7, 10]. The future lies in integrating molecular diagnostics such as PD‐L1 CPS, MSI status, HER2 amplification, and ctDNA to personalize therapy. Immunotherapy, once limited to metastatic disease, is now making inroads in curative settings [10]. Further trials and real‐world data will help refine the optimal combination of regimens, duration, and sequencing strategies for various gastric cancer subtypes.

## 4 Treatment Strategies for Stage IV Gastric Cancer

### 4.1 Systemic Chemotherapy

Stage IV gastric cancer encompasses a broad and heterogeneous group of patients, including those with liver, lung, bone, or peritoneal metastasis, as well as those with bulky nodal disease. Systemic chemotherapy remains the primary modality in unresectable disease, offering palliation and modest survival benefit. In Japan, the SPIRITS trial established the combination of S‐1 and cisplatin (SP) as a standard regimen [32]. Subsequently, the G‐SOX trial demonstrated that SOX (S‐1 and oxaliplatin) was non‐inferior to SP with better safety.

HER2‐targeted therapy is also a critical part of first‐line treatment in HER2‐positive gastric cancer. The ToGA trial demonstrated that adding trastuzumab to chemotherapy significantly prolonged overall survival [33]. Recently, KEYNOTE‐811 investigated pembrolizumab in combination with trastuzumab and chemotherapy, showing a marked improvement in objective response rates, which led to its FDA approval in the first‐line setting [34].

Immune checkpoint inhibitors have changed treatment paradigms. The CheckMate‐649 trial demonstrated improved overall survival with nivolumab added to chemotherapy in patients with PD‐L1 CPS ≥ 5 [4]. In Asia, ATTRACTION‐2 validated nivolumab in the third‐line setting [35]. Pembrolizumab is now used in MSI‐high tumors due to high response rates. Biomarker‐driven strategies are increasingly important.

### 4.2 Treatment for Oligometastasis

Oligometastasis in gastric cancer generally refers to the presence of ≤ 3 metastatic lesions confined to a single organ [36]. In these patients, curative‐intent local therapies may be considered if systemic chemotherapy achieves favorable disease control.

Retrospective studies in Japan have shown 3‐year survival rates exceeding 50% in well‐selected patients undergoing R0 resection after chemotherapy [37, 38]. International efforts, such as the AIO‐FLOT5 (RENAISSANCE) trial, aimed to evaluate the benefit of surgery following induction chemotherapy in oligometastatic disease [39]. Although prematurely terminated due to poor accrual, the study suggested that lack of statistical significance may stem from underpowering rather than ineffectiveness.

Japanese prospective studies have focused on para‐aortic lymph node metastasis as a representative oligometastatic setting. The JCOG0405 phase II trial evaluated S‐1 plus cisplatin followed by D2 gastrectomy with para‐aortic lymph node dissection, achieving an R0 resection rate of 82% and a 5‐year overall survival of 53% [40]. More recently, the JCOG1704 trial investigated neoadjuvant chemotherapy using a triplet regimen (DOS: docetaxel, oxaliplatin, and S‐1) in patients with limited para‐aortic metastases. Preliminary results showed a pathological response rate of 57% and a complete response rate of 24%, supporting the feasibility of conversion‐oriented approaches in selected oligometastatic disease [41].

### 4.3 Conversion Surgery for Initially Unresectable Disease

Conversion surgery involves R0 resection following a favorable response to systemic chemotherapy in patients initially deemed unresectable [42]. Surgical candidacy is reassessed after 3–6 cycles of chemotherapy, often with diagnostic laparoscopy. Studies on conversion surgery in stage IV gastric cancer are summarized in Table 2.

**TABLE 2:** Summary of studies on conversion surgery in stage IV gastric cancer.

Author/trial (year)	Design	No. of patients	Patient cohort	R0 rate (%)	Postop complication (%)	Median OS (months)	References
Du et al. (2019)	Meta‐analysis	1316	Advanced GC (various metastases)	—	24	—	[43]
Ohnuma et al. (2021)	PSM analysis	88	Conversion after induction chemo	85	14	46	[44]
Choe et al. (2019)	PSM analysis	76	Stage IV GC (non‐curable initially)	85	19	44	[45]
Nie et al. (2019)	PSM analysis	190	Conversion in liver metastases	51	—	27	[46]
Yang et al. (2024)	PSM analysis	268	Peritoneal metastasis	80	22	27	[47]
Yoshida et al. (2022)	Retrospective	1206	Conversion after systemic therapy	70	24	37	[48]

Abbreviation: OS, overall survival.

Yoshida et al. proposed a four‐type classification system to guide strategy [49]. Patients in type 2 or 3 who respond to induction therapy can be considered for curative resection. R0 resection rates range between 40% and 60%, with favorable 3‐year survival in responders [50].

Triplet regimens such as DOS or DCS are frequently used, offering higher response rates that facilitate conversion [51]. Extended resections may be necessary depending on the disease extent. Multidisciplinary evaluation and intraoperative decision‐making are essential for optimal outcomes.

The role of postoperative continuation of the preoperative regimen remains unclear. The PRODIGY trial continued S‐1 after DOS and surgery, aiming to reduce recurrence, but prospective evidence on this strategy is limited [10].

Conversion surgery provides a realistic curative approach for selected stage IV patients and continues to evolve through advances in systemic therapy and biomarker‐driven selection. Integration of tools such as MSI status and ctDNA analysis may further refine eligibility in the future [52].

### 4.4 Unresolved Questions and Ongoing JCOG Trials

Despite growing interest in conversion surgery for stage IV gastric cancer, several key questions remain unresolved. These include:

- The optimal timing and duration of induction chemotherapy before surgery.
- Appropriate criteria for selecting conversion candidates.
- Whether surgical intervention truly offers a survival advantage over chemotherapy alone.

To address these gaps, the Japan Clinical Oncology Group (JCOG) has launched several important prospective trials.

JCOG1802 is an ongoing phase III trial targeting patients with limited metastases (e.g., solitary liver or lymph node metastases) who show a favorable response to induction chemotherapy. It aims to determine whether conversion surgery provides a survival benefit over continued systemic therapy in this population [53].

JCOG2301, initiated more recently, represents the first randomized phase III trial specifically designed to evaluate the efficacy of conversion surgery in patients with initially unresectable, extensive stage IVB disease. Eligible patients include those with ≥ 4 liver metastases, para‐aortic lymph node involvement, or peritoneal dissemination. After induction chemotherapy, patients showing a good response are randomized to either continue chemotherapy or undergo conversion gastrectomy with curative intent. The primary endpoint is overall survival, and secondary endpoints include progression‐free survival and recurrence‐free survival. The results of this trial are expected to directly inform clinical practice and potentially establish conversion surgery as a standard approach in selected stage IV patients [54].

Until the outcomes of these trials are available, treatment decisions will continue to rely on retrospective data, multidisciplinary evaluation, and institutional expertise. Nonetheless, these studies signal a pivotal shift toward evidence‐based integration of surgical intervention in metastatic gastric cancer.

## 5 Perioperative Nutrition

### 5.1 Preoperative Nutritional Assessment and Surgical Outcomes

Preoperative malnutrition, sarcopenia, and frailty are highly prevalent in patients with gastric cancer, particularly in older adults, and are strongly associated with adverse postoperative outcomes, including increased morbidity, longer hospitalization, and reduced survival. As gastric cancer often results in poor oral intake and chronic inflammation, nutritional depletion is frequently observed at diagnosis [43].

The Global Leadership Initiative on Malnutrition (GLIM) criteria provide a widely accepted framework for diagnosing malnutrition, incorporating phenotypic indicators such as low body mass index (BMI) and reduced muscle mass (e.g., via CT‐based sarcopenia assessment), along with etiologic factors such as inflammation or decreased intake [44, 45]. Recent prospective studies have validated the prognostic value of GLIM‐defined malnutrition, showing significant associations with longer hospital stays, delayed recovery, and inferior overall survival in gastric cancer patients undergoing gastrectomy [47].

The prognostic nutritional index (PNI)—calculated using serum albumin levels and peripheral lymphocyte count—is also well‐established as a simple, objective tool for perioperative risk stratification. A low PNI has been consistently linked with a higher incidence of infectious complications, poorer tolerance to chemotherapy, and worse long‐term outcomes [46]. Sarcopenia—a key component of malnutrition—has emerged as an independent predictor of postoperative complications and impaired functional recovery. Quantitative muscle indices such as psoas muscle index (PMI) or skeletal muscle index (SMI) assessed on cross‐sectional imaging are increasingly being used to support risk prediction [48, 55].

Given the strong evidence linking poor nutritional status with adverse outcomes, a comprehensive nutritional assessment—including GLIM criteria, PNI, and body composition analysis—should be incorporated into routine preoperative evaluation. Early identification of high‐risk patients enables timely nutritional interventions, such as oral nutritional supplements or immunonutrition, which have shown efficacy in improving perioperative recovery and reducing complication rates [43, 56]. Multidisciplinary collaboration among surgeons, oncologists, and dietitians is essential to optimize nutritional status and surgical outcomes in this vulnerable patient population.

### 5.2 Perioperative Nutritional Therapy and Clinical Trials

Several RCTs in Japan have demonstrated the efficacy of perioperative oral nutritional supplements (ONS) in gastric cancer patients. Miyazaki et al. conducted a phase 3 multicenter open‐label RCT and reported that ONS significantly mitigated postoperative body weight loss following gastrectomy [57]. More recently, Omori et al. conducted a multicenter RCT involving 250 patients and demonstrated that perioperative ONS significantly reduced the incidence of surgical site infections (7.8% vs. 14.2%) and accelerated the recovery of bowel function [58]. These findings support the routine integration of ONS into perioperative care. Moreover, immunonutrition has become an essential component of ERAS protocols, given its potential to modulate immune function and enhance surgical outcomes.

### 5.3 Postoperative Nutritional Disorders and Cachexia

After gastrectomy, patients commonly experience nutritional deficiencies due to reduced gastric capacity, altered digestion, and rapid intestinal transit. These lead to weight loss, anemia (from iron, folic acid, and B12 deficiency), and bone demineralization (due to impaired calcium and vitamin D absorption) [59]. Dumping syndrome, particularly rapid gastric emptying and hypoglycemia, also reduces quality of life. Dietary counseling and regular nutritional follow‐up are essential to mitigate these effects [60].

Cancer cachexia, defined by progressive muscle loss and systemic inflammation, remains a challenge in advanced gastric cancer. Ghrelin agonists such as anamorelin have shown promise in increasing appetite and lean body mass. A meta‐analysis of five trials including 1331 patients demonstrated that anamorelin significantly increased weight and lean body mass [61]. However, its effects on functional outcomes and survival are limited. A study by Tsukuyama et al. noted that early discontinuation of anamorelin correlated with shorter survival, highlighting the need for better supportive interventions [62]. Multidisciplinary models combining nutritional support, exercise, and symptom management are increasingly necessary.

### 5.4 Future Perspectives and Digital Tools

Technological innovations are rapidly transforming perioperative nutritional care for gastric cancer patients. One of the most promising advances is the integration of digital health technologies such as continuous glucose monitoring (CGM), wearable devices, telemedicine, and AI‐based tools [63, 64, 65].

CGM offers a real‐time, dynamic view of glycemic fluctuations, making it particularly useful in detecting late dumping syndrome after gastrectomy [63]. Traditional finger‐stick glucose testing may miss transient but clinically significant hypoglycemic episodes, especially nocturnal ones.

Studies by Shoda et al. have shown that CGM identifies hypoglycemia in more than 80% of patients after Roux‐en‐Y reconstruction after distal gastrectomy or double‐tract reconstruction after proximal gastrectomy, even in patients with no apparent symptoms or normal HbA1c levels [66, 67]. Such fluctuations may contribute to fatigue, dizziness, cognitive decline, and increased cardiovascular risk, especially in elderly patients.

Importantly, CGM allows for personalized dietary counseling. By recognizing individual glycemic patterns, clinicians can recommend tailored interventions such as adjusting meal frequency, composition, and carbohydrate intake. CGM‐guided nutritional care not only improves glycemic stability but also has the potential to enhance quality of life [68].

Beyond CGM, AI and machine learning are increasingly used to analyze dietary patterns and forecast nutritional deficiencies [69]. These technologies support dietitians and clinicians by providing real‐time feedback, automating risk stratification, and suggesting individualized meal plans. When integrated into electronic medical records, these tools facilitate seamless communication across multidisciplinary teams.

Telemedicine has also emerged as an effective way to deliver nutrition consultations and monitor progress, especially in the context of Enhanced Recovery After Surgery (ERAS) protocols. Remote follow‐up enables timely adjustment of nutritional strategies and improves adherence to perioperative guidelines. Coupled with mobile applications and wearable sensors, telemedicine can extend care beyond hospital settings [65, 70].

Although these digital strategies show great promise, challenges remain. These include the need for validation in large clinical trials, addressing disparities in digital literacy among elderly patients, and integrating technologies into existing healthcare systems. Nevertheless, the convergence of CGM, AI, and telehealth in perioperative nutrition represents a major advancement toward truly personalized care in gastric cancer surgery.

## 6 Conclusion

Recent years have witnessed significant progress in multimodal treatment for gastric cancer, including perioperative chemotherapy, conversion surgery, nutritional intervention, and the incorporation of immunotherapy. Robust data from Asian and Western trials continue to shape global standards, while regional variations in surgical and systemic approaches persist.

Stage IV disease, once considered uniformly palliative, is now being approached with curative intent in selected patients through conversion and oligometastasis‐directed strategies. Concurrently, perioperative nutritional care and the use of digital tools such as CGM and AI‐based platforms are enhancing recovery and long‐term outcomes.

Looking ahead, integration of molecular biomarkers, multidisciplinary care, and international collaboration will be key to refining treatment strategies and achieving personalized, evidence‐based care in gastric cancer.

## Author Contributions

**Katsutoshi Shoda:** conceptualization, data curation, formal analysis, funding acquisition, writing – original draft, writing – review and editing. **Yoshihiko Kawaguchi:** writing – original draft. **Suguru Maruyama:** writing – original draft. **Daisuke Ichikawa:** conceptualization, data curation, writing – review and editing.

## Ethics Statement

This study did not require institutional board approval or informed consent because of its retrospective nature and use of anonymized public studies.

## Conflicts of Interest

Daisuke Ichikawa is the Editorial Board Member of AGS. The other authors declare no conflicts of interest.

## Data Availability

All data generated or analyzed during this study are indicated in this article. Further enquiries can be directed to the corresponding author.
